# Review: Nanomaterials for Reactive Oxygen Species Detection and Monitoring in Biological Environments

**DOI:** 10.3389/fchem.2021.728717

**Published:** 2021-09-10

**Authors:** Gabriel T. Huynh, Vidhishri Kesarwani, Julia A. Walker, Jessica E. Frith, Laurence Meagher, Simon R. Corrie

**Affiliations:** ^1^ Department of Chemical Engineering, Monash University, Clayton, VIC, Australia; ^2^ ARC Centre of Excellence in Convergent Bio-Nano Science and Technology, Monash Node, Clayton, VIC, Australia; ^3^ Monash Institute of Medical Engineering, Monash University, Clayton, VIC, Australia; ^4^ Department of Material Science and Engineering, Monash University, Clayton, VIC, Australia; ^5^ ARC Training Centre for Cell and Tissue Engineering Technologies, Monash University, Clayton, VIC, Australia

**Keywords:** ROS—reactive oxygen species, oxygen, nanomaterials, biosensing, bioimaging

## Abstract

Reactive oxygen species (ROS) and dissolved oxygen play key roles across many biological processes, and fluorescent stains and dyes are the primary tools used to quantify these species in vitro. However, spatio-temporal monitoring of ROS and dissolved oxygen in biological systems are challenging due to issues including poor photostability, lack of reversibility, and rapid off-site diffusion. In particular, ROS monitoring is hindered by the short lifetime of ROS molecules and their low abundance. The combination of nanomaterials and fluorescent detection has led to new opportunities for development of imaging probes, sensors, and theranostic products, because the scaffolds lead to improved optical properties, tuneable interactions with cells and media, and ratiometric sensing robust to environmental drift. In this review, we aim to critically assess and highlight recent development in nanosensors and nanomaterials used for the detection of oxygen and ROS in biological systems, and their future potential use as diagnosis tools.

## Introduction

Molecular oxygen has an important impact upon a broad range of biological processes, ranging from its central roles in cellular respiration (Gnaiger et al., 1995; Bartz and Piantadosi, 2010) and enzymatic processes in aerobic organisms (Adeva-Andany et al., 2014) to its activity as a poison to anaerobes (Lungu et al., 2009; Botheju and Bakke, 2011). If oxygen levels fall below cellular requirements, referred to as hypoxia, aerobic cells can experience oxidative stresses due to the increased production of reactive oxygen species (Clanton, 2007; Tafani et al., 2016; Jaitovich and Jourd’heuil, 2017). ROS are a highly reactive oxygen-containing class of small molecules, which can cause a myriad of problems, including irreversible DNA damage (Wiseman and Halliwell, 1996; Yu and Anderson, 1997), protein denaturation (Stadtman and Berlett, 1997) and cell apoptosis (Simon et al., 2000). Examples of ROS molecules include hydrogen peroxide (H_2_O_2_), superoxide anion (O_2_^•-^), hydroxyl radical (^•^OH), singlet oxygen (^1^O_2_), hypochlorite (ClO^−^) and peroxynitrite (ONOO^−^) ions. Hypoxia and subsequent ROS generation play important roles in the progression of many human diseases and impact upon all of the major organ systems, including renal/kidney disease (Heyman et al., 2008; Honda et al., 2019), cardiovascular disease (Giordano, 2005), and neurodegenerative disorders (Emerit et al., 2004; Angelova and Abramov, 2018) such as Alzheimer’s (Huang et al., 2016) and Parkinson’s diseases (Dias et al., 2013; Umeno et al., 2017). While there have been numerous accounts investigating fundamental links between hypoxia, ROS and disease progression (Görlach et al., 2015; Tafani et al., 2016), dynamic and real-time monitoring between the three components in *in vitro* and *in vivo* biological systems has continued to be a challenge.

**FIGURE 1:**
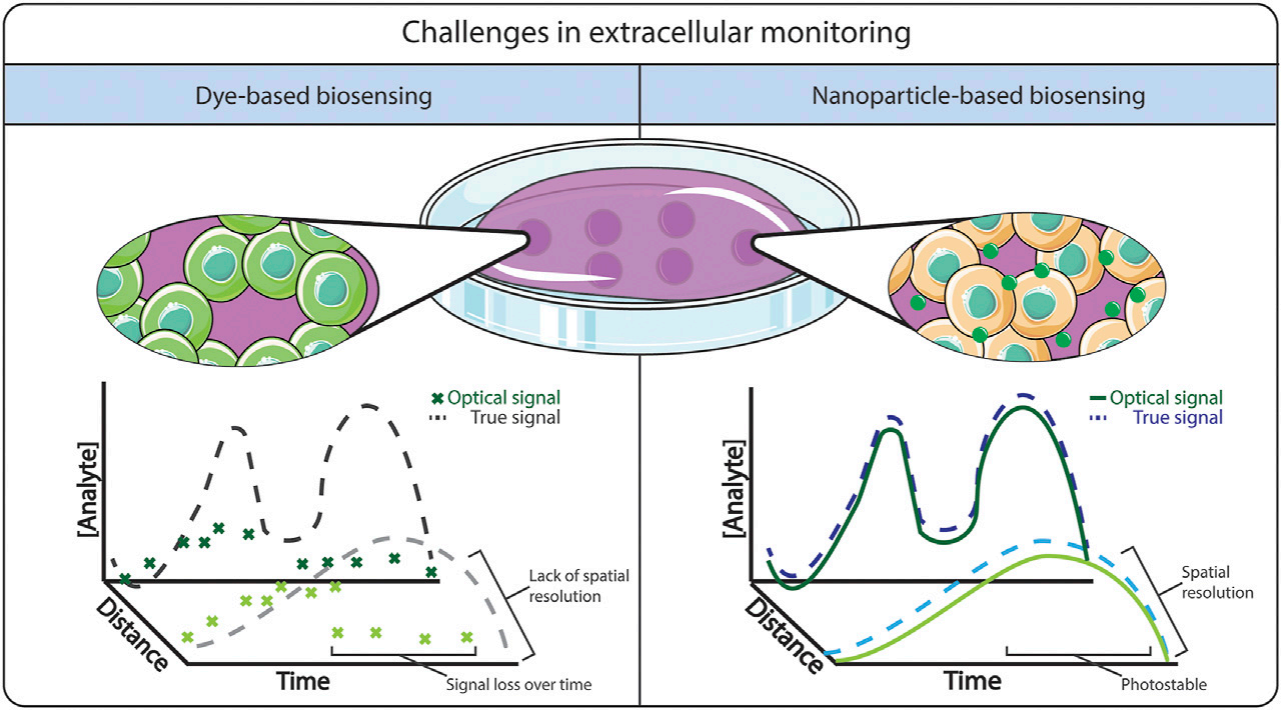
Comparison between free dye-based biosensing versus nanoparticle-based biosensing. The advantages of using nanoparticle-based biosensing is a stable signal from the dye, where it is less prone to photobleaching and dye leaching.

The detection and monitoring of oxygen in biological fluids has been well-developed since the 1950s with the creation of the Clark electrode (Clark et al., 1953; Severinghaus and Astrup, 1986), an electrochemical sensor that dynamically measures the levels of dissolved oxygen. Due to the design of the Clark electrode, the measured oxygen level is relative to the bulk fluid concentration, rather than a localized “point” measurement (Diepart et al., 2010), and hence cannot provide the spatial resolution required for effective monitoring in 2/3D biological systems. The detection of ROS has additional complications, due to their naturally low abundance and short lifetime *in vitro* and *in vivo* (Schmitt et al., 2014). Although there have been reports describing electrochemical devices for the detection and continuous monitoring of ROS *in situ* (Chen et al., 2012)*,* these methods again do not support 2/3D spatial information, and are limited by biological fouling (Wisniewski and Reichert, 2000; Harris et al., 2013; Ruiz-Valdepeñas Montiel et al., 2018). Alternatively, non-invasive fluorescence detection methods have been used extensively to monitor dissolved oxygen and ROS for both *in vitro* and *in vivo* (Zhang et al., 2017b) applications. However, despite high detection sensitivity (Gomes et al., 2005), some fluorescence techniques can suffer from poor fluorescence lifetime/photostability (Eggeling et al., 1999), lack of specificity (Wardman, 2007), and issues related to high background to signal ratio in biological tissue (Monici, 2005). These drawbacks are particularly obvious when the fluorophore is free in solution (O’Riordan et al., 2005) in comparison to when they are encapsulated within a matrix, as illustrated in Figure 1. As a result, there remains a need to develop nanomaterial encapsulation strategies to detect and quantify oxygen and ROS in 2D and 3D biological systems.

The blending of luminescent molecules with nanomaterials shows great promise to support dynamic spatio-temporal monitoring within 2D and 3D cellular systems, as described schematically in Figure 1. Due to their small size and the ability to readily modify physical and chemical properties (Kairdolf et al., 2017), nanoparticles have been shown to improve the optical properties of fluorescence molecules (Koren et al., 2012) by increasing fluorescence intensity and minimising photobleaching (Koo Lee et al., 2009; Lee and Kopelman, 2009). The ability to tune their physio-chemical properties allows tailoring imaging probes in terms of cell permeability, reduced toxicity, improved solubility, and minimizing off-site diffusion to allow spatio-temporal monitoring. One key example is the ongoing development of optical glucose biosensors, whereby the incorporation of nanomaterials such as carbon nanotubes (Barone et al., 2005) and graphene (Shehab et al., 2017), or using enzymes encapsulated within a highly permeable polymeric microsphere (Soda et al., 2018) have been shown to improve the responsiveness of the sensors and could enable monitoring in tissue locations not accessible to electrochemical sensors. This approach has also been applied for the improvement of existing oxygen and ROS sensors. For example, the development of oxygen and ROS optode nanosensors was based on the miniaturization of ion-selective electrodes (Hopf and Hunt, 1994; Wolfbeis, 2015a). Ruckh and Clark (2014) discussed the challenges in adopting nanosensors as a feasible alternative to existing technologies, highlighting that they need to display: 1) dynamic reversibility and fast response time; 2) high analyte selectivity and sensitivity; and 3) good biocompatibility within the biological system.

In this review, we aim to provide a summary of current optical techniques for both oxygen and ROS detection and their current limitations, before discussing recent advances in optical oxygen and ROS nanosensors in 2D and 3D systems, and finally discussing potential alternative methods for dynamic and deep-tissue imaging of ROS. As this review will focus on optical techniques, we recommend the review by Seenivasan et al. (2017), for a thorough description of electrochemical nanosensors for ROS detection.

## Brief Overview of ROS Generation *in situ*

The link between oxygen, hypoxia and ROS generation has been widely reported, whereby endogenous ROS production has been primarily associated with the mitochondria (Chandel et al., 2000; Li et al., 2013). Superoxide anions (O_2_^•-^) are produced within the inner membrane of the mitochondria from two protein complexes: complex I and complex III. During the generation of adenosine triphosphate (ATP), known as oxidative phosphorylation, electrons are transported through the mitochondria via the electron transport chain. Leakages in complex I and complex III cause some electrons to “leak” from the transport chain, to react with nearby oxygen molecules, producing O_2_^•-^. Under normal oxygenation conditions, approximately 1–2% of electrons transported within the mitochondria generate superoxides (Solaini et al., 2010). These superoxide anions are short-lived and are converted into H_2_O_2,_ when O_2_^•-^ binds to either superoxide dismutase 1 (SOD1) or superoxide dismutase 2 (SOD2).

**FIGURE 2:**
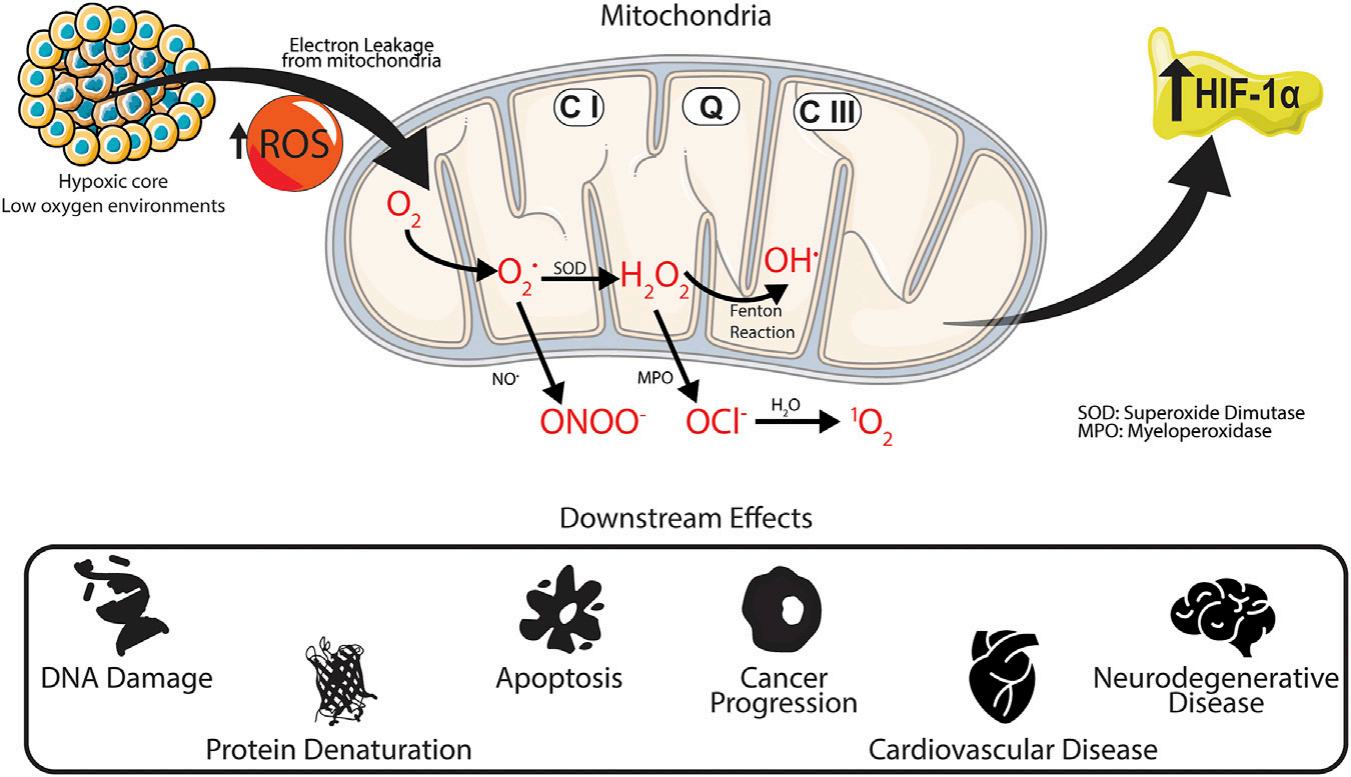
Downstream effects of hypoxia *in situ*. Under low oxygen environments production of superoxides from complex III increases within the mitochondria. The superoxide then reacts to form different ROS products, increasing the upregulation of HIF-1α, which has been linked to various intracellular and extracellular damage associated with several physiological and neurological diseases.

Under low oxygen environments, ROS production in complex III at the Q_0_ site increases (Bell et al., 2007). This in turn increases the number of superoxide species released into the intermembrane space of the mitochondria which are reduced to hydrogen peroxide and escape into the cytosol (Guzy et al., 2005). Transcription factors known as hypoxia-inducible factors (HIF) are expressed and help mediate cellular survival and activity. HIF-1α is the main HIF transcription factor associated with hypoxia and the subsequent cellular response. HIF-1α expression increases under hypoxic conditions (Jiang et al., 1996) and is further stabilized by the presence of ROS. Work conducted by Guzy et al. (2005) *.* established that H_2_O_2_ levels increase under low oxygen environments, whereby increased electron transport in complex III helps stabilize HIF-1α *in vitro*. Additionally, overexpression of HIF-1α has been associated with numerous cancers, including breast (Kimbro and Simons, 2006; Gilkes and Semenza, 2013), prostate (Zhong et al., 1998; Kimbro and Simons, 2006), lung (Volm and Koomägi, 2000), and ovarian cancer (Wong et al., 2003); and neurodegenerative conditions (Merelli et al., 2018), such as Alzheimer’s (Ogunshola and Antoniou, 2009) and Parkinson’s diseases (Dias et al., 2013). Elevated levels of ROS have been linked to DNA damage at the transcriptome level through DNA methylation (Franco et al., 2008; Kietzmann et al., 2017), apoptosis induction though cell signaling and the activation of tumor necrosis factor (TNR) receptors (Simon et al., 2000; Redza-Dutordoir and Averill-Bates, 2016), and protein oxidation leading to denaturation (Stadtman and Berlett, 1997). The relationship between hypoxia, overproduction of ROS and the stabilization of HIF-1α is shown in Figure 2 **.**

ROS generation is a fundamental process that occurs naturally *in situ* and plays key roles in regulating cell fate decisions (Ray et al., 2012), such as cell proliferation, differentiation and survival, as well as having regulatory effects on the anti-inflammatory response as a cell signaling molecule. The presence of antioxidant compounds help minimize ROS levels (Panieri and Santoro, 2016) and facilitate the maintenance of homeostasis. For example, peroxidases, such as glutathione peroxidase and catalase present within cells can reduce levels of H_2_O_2_ by reduction to water. In addition, cytochrome c, a hemeprotein located within the inner membrane of the mitochondria has been shown to oxidise O_2_^•-^ back into O_2_, while reducing H_2_O_2_ back to ^•^OH. Other sources of endogenous ROS generation outside the mitochondria include xanthine oxidase, which is used in the oxidation of hypoxanthine and xanthine into uric acid, with H_2_O_2_ as a by-product; and myeloperoxidase, which promotes the production of OCl^−^ during immune responses.

## Optical Dyes Currently Used for Detection and Monitoring of Oxygen and ROS Species

Fluorescence and phosphorescence molecular probes have a well-established chemistry and are widely used to detect oxygen and ROS *in vitro* (Yoshihara et al., 2017; Zhang et al., 2018) and *in vivo* (Wilson and Cerniglia, 1992; Stepinac et al., 2005; Baudry et al., 2008; Hall et al., 2012; Huntosova et al., 2014; Huntosova et al., 2017). In addition they have high analyte sensitivity, low cost, and a fast response time, enabling rapid, spatially resolved measurements (Terai and Nagano, 2008; Wolfbeis, 2015a)*.* These optical indicators are explicitly developed for one analyte only and are selective towards specific reactions, the products of which are used for quantitative signal analysis, either as a change in luminescence intensity (Zhang et al., 2016), or as a red/blue shift in emission spectra (Pap et al., 2000; Drummen et al., 2004; Liu et al., 2014). Most commonly available oxygen and ROS indicators report changes in luminescence intensity relative to the target analyte concentration. While these methods allow for easy and rapid detection, they required a necessary baseline correction as they are prone to external environmental factors and instrumentation errors, which affects the quantitative analysis. On the other hand, the measurement of changes in peak wavelength provides better accuracy as it does not suffer from the same limitations as measurements of fluorescence intensity (Zhu et al., 2017; Islam et al., 2019). Fluorescence lifetime microscopy (FLIM) has been recently used for monitoring spatio-temporal changes within biological systems (Datta et al., 2020). Unlike steady-state fluorescent detection techniques, where the concentration of dye and the intensity of the light source can affect analyte quantification, FLIM allows for time-resolved monitoring of fluorescence decay of fluorophores, which is unaffected by the above factors (Chang et al., 2007). As the fluorescence lifetime is highly dependent on the microenvironment, FLIM has been used for the detection of ROS levels *in vitro* (Bilan et al., 2013; Balke et al., 2018). While fluorescence has been beneficial for the detection of oxygen and ROS in biological studies, many of the dyes available fluoresce in the visible light region (400–500 nm), limiting their applications because cells and tissues autofluorescence within the same wavelength range. Consequently, there has been growing interest in the development of dyes which fluoresce within the red/near-infrared region (>700 nm) (Li and Wang, 2018).

Metal-ligand complexes, such as metal porphyrins, are commonly used in the development of reversible optical oxygen sensors, including ruthenium (II), platinum (II), palladium (II) and iridium (II) complexes. Ruthenium-based porphyrins have been used as fluorescent oxygen-sensitive dyes, due to their high photostability and quantum yield, and fast response time. [Ru(dpp)_3_]^+2^, [Ru(phen)_3_]^+2^, and [Ru(bpy)_3_]^+2^ have been used extensively in the development of commonly available fiber optical oxygen sensors (MacCraith et al., 1993; Draxler et al., 1995; Choi and Xiao, 2000) for *in vitro* (Choi et al., 2012) and *in vivo* (Paxian et al., 2004; Zhang et al., 2017b; Guan et al., 2020) imaging of oxygen tension in biological systems*.* One of the main challenges with these dyes is their hydrophobicity and poor cellular uptake (Yoshihara et al., 2017), which limits their direct use for dynamic monitoring in aqueous solutions. These limitations can be mediated somewhat by chemical modification (Khan et al., 2015), such as the addition of hydrophilic functional groups. In addition, due to the visible light fluorescent properties of ruthenium-based dyes, phosphorescence oxygen-responsive dyes have been used to circumvent issues with tissue autofluorescence. These phosphorescence dyes, such as platinum (II) and palladium (II) porphyrins are advantageous in that they can be used for deep-tissue imaging, due to their large Stokes shift emitting within the near-infrared region (Borisov et al., 2008; Pereira et al., 2017). In addition, the use of these dyes has been reported for imaging hypoxia during tumor progression *in vivo* following encapsulation (Zhao et al., 2015; Lv et al., 2018). A summary of common oxygen-responsive fluorophores can be found in Table 1. A more in-depth review on the various types of oxygen-responsive dyes can be found in the review article authored by Quaranta et al. (2012).

**TABLE 1:** Summary of commonly used oxygen-responsive optical dyes for monitoring oxygen levels in biological systems.

Optical dye	Peak λ_ex_ (nm) |	Optical mechanism	Reversible or irreversible	Intensity/Spectra change upon interaction
	Peak λ_em_ (nm)			
tris (4,7-diphenyl-1,10-phenanthroline) ruthenium(II) [Ru(dpp)_3_]^+2^	463 | 618	Fluorescence	Reversible	Emission intensity change
tris (1,10-phenanthroline) ruthenium(II) [Ru(phen)_3_]^+2^	444 | 596	Fluorescence	Reversible	Emission intensity change
tris (2,2′-bipyridyl) ruthenium(II) [Ru(bpy)_3_]^+2^	450 | 630	Fluorescence	Reversible	Emission intensity change
Platinum (II) octaethylporphyrin PtOEP	382/536 | 649	Phosphorescence	Reversible	Emission intensity change
Platinum (II) 5, 10, 15, 20-tetrakis-(2,3,4,5,6-pentafluorophenyl)-porphyrin PtTFPP	390 | 647/710	Phosphorescence	Reversible	Emission intensity change
Platinum (II) tetrakis-(4-carboxyphenyl)-porphyrin PtTCPP	402 | 675	Phosphorescence	Reversible	Emission intensity change
Palladium (II) octaethylporphyrin PdOEP	546 | 670	Phosphorescence	Reversible	Emission intensity change
Palladium (II) 5, 10, 15, 20-tetrakis-(2,3,4,5,6-pentafluorophenyl)-porphyrin PdTFPP	406 | 738	Phosphorescence	Reversible	Emission intensity change
Palladium (II) tetrakis-(4-carboxyphenyl)-porphyrin PdTCPP	418 | 700	Phosphorescence	Reversible	Emission intensity change

With respect to ROS responsive dyes, there is also a range of commercially available stains, as listed in Table 2. Fluorescence quantification of *in situ* ROS concentration has been used extensively due to the high sensitivity and rapid response time. However, these dyes are limited by low specificity and cross-reactivity with other ROS species (Henderson and Chappell, 1993; Crow, 1997; Kooy et al., 1997; Ischiropoulos et al., 1999; Wrona et al., 2005), and that the chemistries used are not reversible so these dyes cannot be applied to track target molecules over time. These issues are connected to the chemical structure of the dye, where the fluorophore undergoes an oxidation reaction in the presence of ROS, which changes the fluorescence properties. As this oxidation reaction is non-selective and irreversible, continuous spatio-temporal monitoring of ROS levels *in situ* remains a challenge in 2D and 3D environments.

**TABLE 2:** Common commercially available fluorescence for ROS detections in biological systems.

Stain	Target species	Peak λ_ex_ (nm) | Peak λ_em_ (nm)	Optical mechanism	Reversible or irreversible	Intensity/Spectra change upon interaction
Dichlorofluorescein diacetate	Non-specific	488 | 530	Fluorescence	Irreversible	Emission intensity change
Dihydrorhodamine 123	Non-specific	505 | 534	Fluorescence	Irreversible	Emission intensity change
CellROX® Green/Orange/Deep Red	Non-specific	485 | 520	Fluorescence	Irreversible	Emission intensity change
		545 | 565			
		644 | 665			
coumarin-3-carboxylic acid	^•^OH	350 | 395	Fluorescence	Irreversible	Emission intensity change
Singlet Oxygen Sensor Green Reagent	^1^O_2_	504 | 525	Fluorescence	Irreversible	Emission intensity change
MitoSOX® Red Mitochondrial Superoxide	O_2_^•-^	510 | 580	Fluorescence	Irreversible	Emission intensity change
Dihydroethidium	O_2_^•-^	535 | 610	Fluorescence	Irreversible	Emission intensity change
Amplex® Red hydrogen peroxide	^•^OH	571 | 585	Fluorescence	Irreversible	Emission intensity change
Aminophenyl fluorescein	^•^OH, ClO^−^, ONOO^−^	490 | 515	Fluorescence	Irreversible	Emission intensity change
Hydroxyphenyl fluorescein	^•^OH, ONOO^−^	490 | 515	Fluorescence	Irreversible	Emission intensity change

## Optical Nanosensors for Detection and Monitoring of Oxygen and ROS *in vitro* and *in vivo*

**FIGURE 3:**
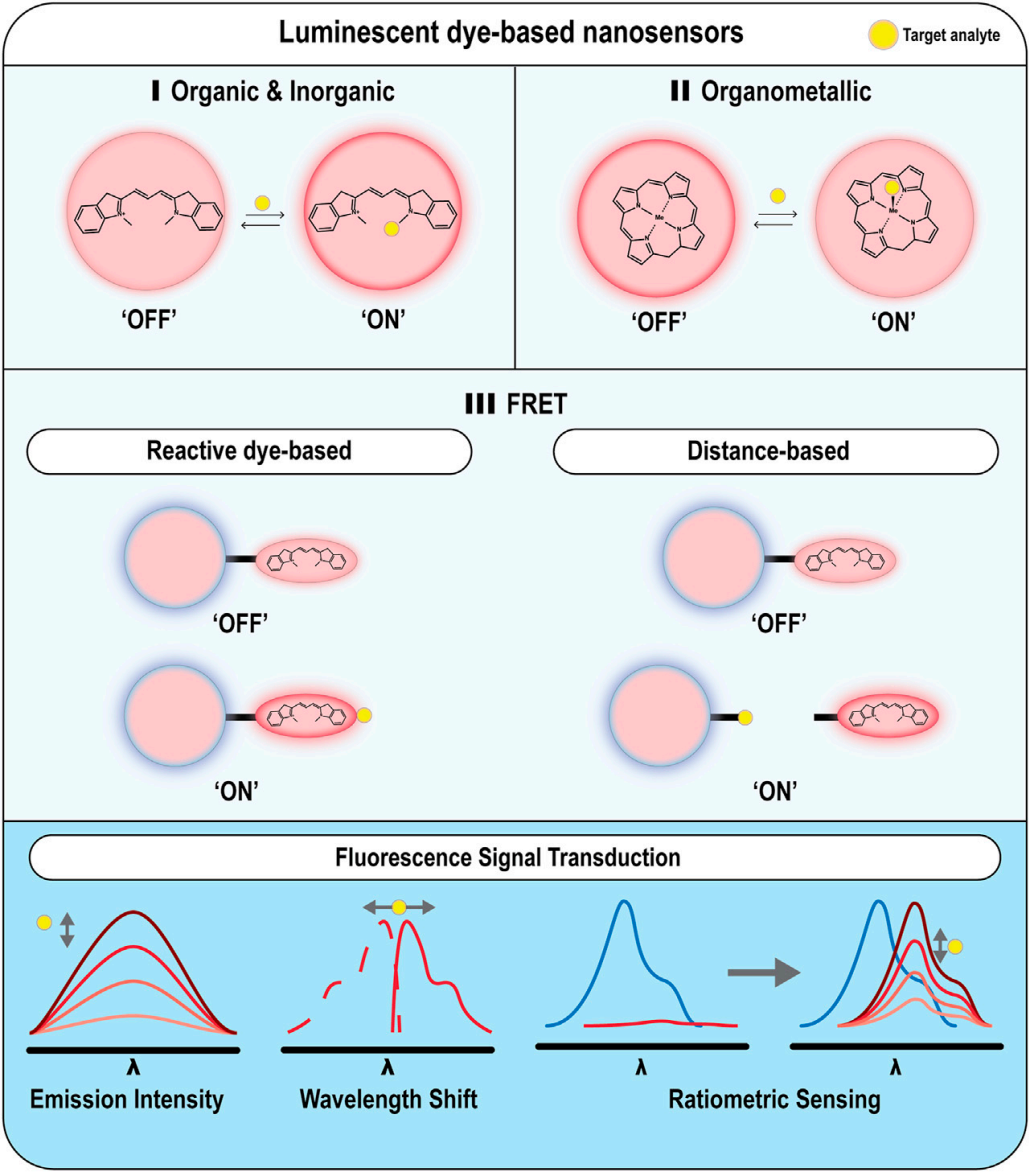
Examples of different nanoparticle-based optical techniques for monitoring oxygen and ROS. Nanoparticles can be either functionalized with **(A)** organic or inorganic dyes for monitoring ROS **(B)** organometallic dyes for monitoring O_2_ levels, or **(C)** FRET pairs for monitoring ROS species.

Encapsulation of oxygen and ROS-sensitive dyes within nanoparticle scaffolds has improved the stability and signal intensity for imaging purposes. While there is no definitive explanation on the mechanism for the enhancement of fluorescence emission once dyes are encapsulated within nanomaterials, it has been suggested that it could be due to environmental/matrix-dependent fluorescence lifetime changes (Muddana et al., 2009; Terrones et al., 2017), or fluorophore protection from non-specific protein interactions and/or external environmental factors (Wolfbeis, 2015b; Reisch and Klymchenko, 2016). As discussed by Aylott (2003), Kopelman (Lee et al., 2009), and Wolfbeis (2015b), the main advantages of fluorescence/phosphorescence nanoparticles over their free dye counterparts are: 1) the ability to encapsulate within a biocompatible matrix which minimizes toxic effects from the free dye; 2) tuneable cellular uptake, which can be further enhanced with nanoparticle surface modification; 3) a reduction in optical interference from protein binding or external environmental effects; 4) improved photostability and signal intensity due to higher dye loading; 5) *in vivo* calibration is near-identical when applied *in vitro;* and 6) opportunities for ratiometric or multiplex sensing with the additional dye loading. A wide variety of dyes with different signal transduction mechanisms have been incorporated into nanomaterials, as described in Figure 3. Importantly, while changes in fluorescence intensity are most often conceptually linked to imaging via microscopy etc., it is important to note that a wide array of both imaging and spectroscopic approaches can also be captured in modern microscopy experiments (Arandian et al., 2019), and common spectral approaches involve wavelength-shifting species and ratiometric sensing, including Förster Resonance Energy Transfer (FRET) approaches.

Work by Kim et al. (2010b) showed that encapsulation of dichlorofluorescein acetate, a non-selective ROS indicator, into a silica scaffold improved the selectivity of the dye to H_2_O_2_, relative to other ROS molecules. In addition, the incorporation of fluorophores within nanoparticle scaffolds can help mitigate undesirable traits associated with the dye, such as poor cell permeability. (Ruiz-González et al., 2017) demonstrated improvements for Singlet Oxygen Sensor Green (SOSG), a commercially available stain for ^1^O_2,_ by conjugation onto a polyacrylamide nanoparticle surface. SOSG is a cell impermeable stain (Prasad et al., 2018), whose fluorescence properties are red-shifted in protein-containing solutions (Gollmer et al., 2011), limiting application for real-time monitoring in *in vitro* cell cultures. However, by encapsulating the dye into a polyacrylamide scaffold, the authors were able to stabilise the fluorescence wavelength shift associated with SOSG in protein serum. In addition, by encapsulating SOSG within the scaffold, good cell permeability was demonstrated. In this study, *E. coli* was use as a model organism, in which the nanoprobes were readily internalized, allowing monitoring of intercellular ^1^O_2_.

While incorporating optical dyes within nanosensors has helped with performance and selectivity, there are still practical limitations of single-labelled nanosensors. One key issue is signal variation and environmental drift over prolonged time periods, such as from photobleaching, light scattering effects, or variations in nanosensor concentration within a field of view or between experiments (Bigdeli et al., 2019). To minimize these effects, dual-labelled or ratiometric fluorescent nanosensors have been developed (Doussineau et al., 2010). The design of these ratiometric nanosensors involves the addition of a secondary analyte-insensitive fluorophore to act as an internal reference and minimize erroneous measurements, either from signal drift or external environmental factors, while improving the signal-to-noise ratio (Huang et al., 2018; Bigdeli et al., 2019). Additionally, it has been reported that the particle concentration does not affect the signal output within a broad range (Robinson et al., 2018), making ratiometric sensors an attractive alternative. These sensors comprise two fluorophores, where both dyes are encapsulated separately within a nanoparticle scaffold, or one fluorophore is chemically attached onto the surface of a responsive nanoparticle (i.e. FRET-based nanosensors).

### Fluorescent and Phosphorescent Ratiometric Nanosensors for Oxygen and ROS Detection

One of the earliest accounts of ratiometric nanosensors for ROS (King and Kopelman, 2003; Kim et al., 2010b) and oxygen (Xu et al., 2001; Cao et al., 2004; Lee et al., 2010) sensing was from the Kopelman group, referred to as PEBBLE (probes encapsulated by biologically localized embedding) nanosensors. PEBBLEs are a class of optodes and were developed as an alternative to ion-selective electrodes (ISEs). A key advantage is that the miniaturization of the technology allows for non-invasive and simplified analyte detection (Xie and Bakker, 2015). While ion-selective electrodes depend on the immobilization of ionophores within a polymeric membrane to transduce an electrical signal, optodes encapsulate an ionophore and a chromophore within a lipophilic nanoparticle for optical signal transduction. The key advantage of PEBBLEs and similar nanoparticle-based sensors is that they permit high spatial and temporal resolution within cell culture systems, whilst ISEs are limited to changes surrounding the electrode (Wolfbeis, 2008). While the Bakker (Xie et al., 2014; Jarolímová et al., 2016) and Suzuki (Soda et al., 2018) groups have developed optodes for biological sensing, the Kopelman group and their associated PEBBLE design will be used as a key example in this review, due to their exemplification of a wide array of chemical sensors for biological monitoring of different analytes (Clark et al., 1999; Xu et al., 2001; Cao et al., 2004; Dubach et al., 2010).

One example from the Kopelman group is the development of optode-based nanosensors to monitor oxygen (Xu et al., 2001), where Xu et al. (2001) encapsulated an oxygen sensitive dye [Ru(dpp)_3_]^+2^ and Oregon Green as a reference fluorophore to monitor gaseous oxygen concentration and intracellular oxygen levels within rat C6 glioma cells. This study demonstrated the key sensor characteristics ideal for biosensing applications: good reversibility and dynamic range, while displaying minimal dye leaching and photobleaching and is therefore an approach that could be further used to provide valuable information on key biological processes related to intracellular oxygen. Additionally, King and Kopelman (2003) developed a polyacrylamide-based ^•^OH nanosensor that utilized coumarin-3-carboxylic acid as the ROS-responsive fluorophore, and Texas Red as the reference dye. In the presence of ^•^OH, the non-fluorescence coumarin dye was converted to a fluorescent 7-hydroxycoumarin compound. However, some reports suggest that the fluorescence signal of 7-hydroxycoumarin is pH-dependent (Fink and Koehler, 1970) which coupled with the irreversible nature of the oxidation reaction, may limit this system as an accurate and dynamic sensor. Additionally, the selectivity of the nanoprobe against other ROS molecules was not investigated. To further investigate the selectivity of coumarin-3-carboxylic acid nanosensors, outside work by Liu et al. (2016) developed a similar silica-based sensor in which rhodamine was used as the reference dye. By comparing the performance of the probe against other ROS molecules and metal ions, it was confirmed that this sensor was highly selective towards ^•^OH and could be used to tracked ^•^OH generation within HeLa cells. Despite the promise of this work, the authors noted that due to the limitations of dynamic ^•^OH fluorophores, the sensor could be limited to monitoring changes in ^•^OH homeostasis through continuous addition of new nanosensors.

Ratiometric nanosensors have also been investigated for imaging oxygen levels *in vivo*, highlighting the potential for deep-tissue hypoxia imaging. Napp et al. (2011) used near-infrared ratiometric oxygen nanosensors to image dynamic tissue deoxygenation in real time. Due to the autofluorescence of tissue, near-infrared optical indicators have been used to help minimize background interference when quantifying oxygen levels. Here, polystyrene nanoparticles containing palladium porphyrin and a reference dye were used, where the large Stokes shift of the metal porphyrin was used to report dynamic changes in oxygen levels and distribution. This could be measured within live and recently-culled mouse tissue *in vivo* with minimal background tissue interference and showed the potential for such an approach to image changing oxygen levels within tumor-bearing mice. Similarly, the McShane group developed a ratiometric oxygen microsensor to monitor different oxygen levels in solution. Collier et al. (2011) combined a carboxyl-functionalized platinum porphyrin and a near-infrared quantum dot onto an amine-functionalized silica scaffold as a ratiometric sensor. The authors demonstrated the functionality of their sensor as a near-infrared sensor at different O_2_ gas concentrations and were able to show the dynamic range, whilst demonstrating the photostability and dynamic functionality. While this sensor was not tested in a biological system, the authors highlighted potential future applications where near-infrared sensing would be advantageous, including monitoring oxygen levels *in vivo*, or within hypoxic tumor microenvironments (Harris, 2002). In fact, the McShane group has developed a wide array of oxygen nanosensors as a platform for the development of highly specific enzymatic dynamic biosensors, such as lactate (Biswas et al., 2017) and glucose (Brown et al., 2004; Unruh et al., 2015; Bornhoeft et al., 2017) for *in vivo* applications, demonstrating their utility in alternative sensing applications.

### Förster Resonance Energy Transfer Based Nanosensors for ROS Detection

Förster Resonance Energy Transfer (FRET) is a photoelectric phenomenon between two fluorescing species that has been used as a method for detection of ROS levels *in vitro* and *in vivo.* FRET is dependent on the distance and energy transfer between two chromophores, commonly known as the donor and the acceptor (Rowland et al., 2015). If the distance between the two chromophores is small, approximately <10 nm (Medintz and Hildebrandt, 2013) and the donor chromophore is excited, its corresponding fluorescence spectrum is quenched as the energy (“ON”) is transferred to the acceptor chromophore, allowing it to fluoresce to produce a fluorescence signal or spectra. However, if the distance between the two chromophores is larger than 10 nm, the emission spectrum of the donor chromophore is no longer quenched (“OFF”). The selection of the fluorophores depends on the degree of spectral overlap between them. A review by Wu et al. (2020) summarised the standard designs for FRET biosensors (Wu et al., 2020). To further categorize the nanosensor designs, for this review we will label them as: 1) distance-based FRET sensors, where the distance between the two fluorophores can be changed; or 2) reactive fluorophore-based FRET sensors for ROS detection (Figure 3).

Distance-based FRET sensors depend on manipulating the distance between the donor and the acceptor molecules, where the acceptor fluorophore quenches the donor fluorescence signal. Here, the two chromophores are coupled onto an analyte-sensitive linker molecule, where the distance between the two chromophores is small. In the presence of the target analyte, the linker molecule connecting the donor and acceptor molecules undergoes a conformational change, either by unwinding/extending the distance between the fluorophores or the linker is cleaved. In either event, the distance between the fluorophores increases such that the donor fluorescence is no longer quenched and direct quantification of ROS levels can be determined by the change of fluorescence intensity. Examples of common ROS-sensitive reactive groups are thioketals, phenylboronic acids/esters, vinyldithioethers, or diselenide bonds. For a more detailed summary of these groups, their mechanism and their respective applications in ROS-based therapy, we direct readers to an extensive review by Tapeinos and Pandit (2016).

Diselenide bonds are an attractive candidate for developing ROS-selective linkers as they are stable under physiological conditions while being easily oxidized by H_2_O_2_ (Deepagan et al., 2016; Tapeinos and Pandit, 2016; Deng et al., 2017). Recently, Deepagan et al. (2018) developed a FRET nanosensor, which used the diselenide bond to control the sensor’s responsiveness. Here, gold nanoparticles were decorated with fluorescein via a diselenide linker. Due to the length of the linker, the fluorescence signal from the fluorescein dye was quenched by energy transfer from the gold nanoparticle. A strong fluorescence signal was obtained from cleaved fluorescein when the sensor was used to monitor H_2_O_2_ in macrophages, allowing the detection of ROS *in vitro*. However, the use of diselenide bonds has not been used for further development of ROS-selective nanosensors, despite having been reported in the design for H_2_O_2_-mediated drug delivery (Deepagan et al., 2016) and gene transfection (Deng et al., 2017).

Phenylboronic acids/esters are reactive groups that have been explored for FRET-based nanosensors, due to the presence of H_2_O_2_-specific cleavage sites. This oxidation reaction (Kuivila, 1954) is highly specific to the nucleophilic attack of H_2_O_2_, where other ROS molecules are unable to break the phenylboronic ester bond (Song et al., 2014a). Feng et al. (2017) developed a polymeric self-assembled FRET nanosensor to detect H_2_O_2_ using a phenylboronic ester linkage. An amphiphilic polymer was used as a self-assembled scaffold, where the fluorophore pair was 7-hydroxycourmain-3-carboxylic acid, and 4-carboxyl-3-fluorophenylboronic acid-functionalized Alizarin Red S. Within the self-assembled scaffold, the fluorescence signal from hydroxycoumarin was quenched by Alizarin Red S. Due to the presence of the boronic acid linker conjugated onto the Alizarin Red S, the fluorescence ratio between 7-hydroxycourmain-3-carboxylic acid and Alizarin Red S could be used to detect H_2_O_2_ in biological solutions, as the linker was selectively cleaved in the presence of H_2_O_2._

In contrast to distance-based FRET sensors, reactive fluorophore-based FRET sensors are dependent on the reactivity of the acceptor fluorophore with the target analyte. Depending on the acceptor fluorophore and the design of the FRET pair, the sensor can be either be “ON” (i.e*.* the donor fluorophore is quenched) or “OFF” (i.e. the acceptor fluorophore is quenched) prior to interaction with the targeted analyte. Irrespective of the design of the FRET design, when the target analyte interacts with the acceptor fluorophore, there is a change in the optical output signal and the analyte can be quantified by the change in fluorescence intensity of the acceptor fluorophore. Unlike the distance-based FRET design discussed above, this strategy is dependent on the sensitivity of the acceptor molecule reaction with the target molecule. Nanosensors of this design typically use metallic nanoparticles, which are mostly used as donor chromophores such as quantum dots (Cardoso Dos Santos et al., 2020) or gold nanoparticles (Cao et al., 2011). These metallic nanoparticles display great photostability over prolonged periods of time, and possess size-dependent fluorescence properties, allowing them to be used as tunable alternatives to conventional imaging probes (Alivisatos et al., 2005; Medintz et al., 2005; Resch-Genger et al., 2008).

One recent example of a reactive fluorophore-based FRET sensor is an infrared FRET-based nanosensor, developed by Li et al. (2020a) to monitor the progression of ONOO^−^ as an early detection for traumatic brain injury. This nanosensor was developed to attempt to overcome the current challenges associated with real-time monitoring of traumatic brain injury, such as computed tomography imaging, but are limited to physical/anatomic information and cannot provide information on relevant biochemical events, such as the levels of ONOO^−^ and its connection with brain injury. For detecting ONOO^−^, Ag_2_S quantum dots were functionalized with a ONOO^−^reactive dye A1094 along with a targeting peptide for high specificity towards the area of interest present within brain injuries. Due to the overlapping spectra of A1094 and the quantum dot, in the absence of ONOO^−^, the fluorescence signal of the quantum dot was quenched as a consequence of absorption of the emitted light by the neighboring dye. However, once the dye was oxidized by ONOO^−^, the signal from the quantum dot was no longer quenched, allowing for direct quantification of the targeted analyte. When used for an *in vivo* assessment of ONOO^−^ production during induced traumatic brain damage, they were able to image the generation of ONOO^−^ in real time, demonstrating the dynamic functionality of their sensors.

A study by Fang et al. (2020), elaborated on the development of a ratiometric fluorescent nanoprobe for the detection of highly reactive oxygen species. The nanoprobe used an oxidation-regulated FRET generated by gold nanoclusters coupled with *o*-phenylenediamine (OPD), a compound that is specifically oxidised by a hydroxyl radical to form the fluorescence compound: 2,3-diaminophenazine (DAP). Although gold nanoclusters can be directly oxidised by reactive oxygen species, including ClO^−^, ONOO^−^ and ^•^OH, yielding a change in fluorescence signal, the reaction is not specific to hydroxyl radicals, preventing its use as a highly selective ROS nanoprobe (Li et al., 2017). Herein, the group fabricated gold nanoclusters through a one-pot, ecofriendly approach that was used in conjunction with OPD to selectively detect hydroxyl radicals. The gold nanoclusters demonstrated high fluorescence intensity that was stable over a range of physiological pH. It should be noted, however, unlike other FRET-based optical nanosensors, the fabricated gold nanoclusters were not tethered to OPD. Nonetheless, the nanosensors successfully demonstrated specificity towards hydroxyl radicals upon introduction of OPD when tested against a variety of other ROS, reactive nitrogen species (RNS), and metal ions, that were being examined, signifying the usability.

### Environmental Sensitive Nanomaterials for ROS Monitoring and Detection

#### Single-Walled Carbon Nanotubes for Multiplex Sensing

Single-walled carbon nanotubes (SWCNTs) have been recently explored as possible nanomaterials for biosensing applications, where their chirality, electronic structure and photophysical behavior enables them to emit fluorescence signals within the near-infrared region (900–1,500 nm) which can be used for deep tissue imaging (Kruss et al., 2013). The main advantage of SWNCTs is that they do not photobleach, making them advantageous as compared to fluorescently labelled nanomaterial scaffolds (Hartschuh et al., 2005). However, one limitation of carbon nanotubes is their hydrophobicity and poor biocompatibility, which requires additional surface modifications to be made, including coating with lipids or DNA strands (Karousis et al., 2010) to improve their biocompatibility such that they are more acceptable for use in biomedical applications.

**FIGURE 4:**
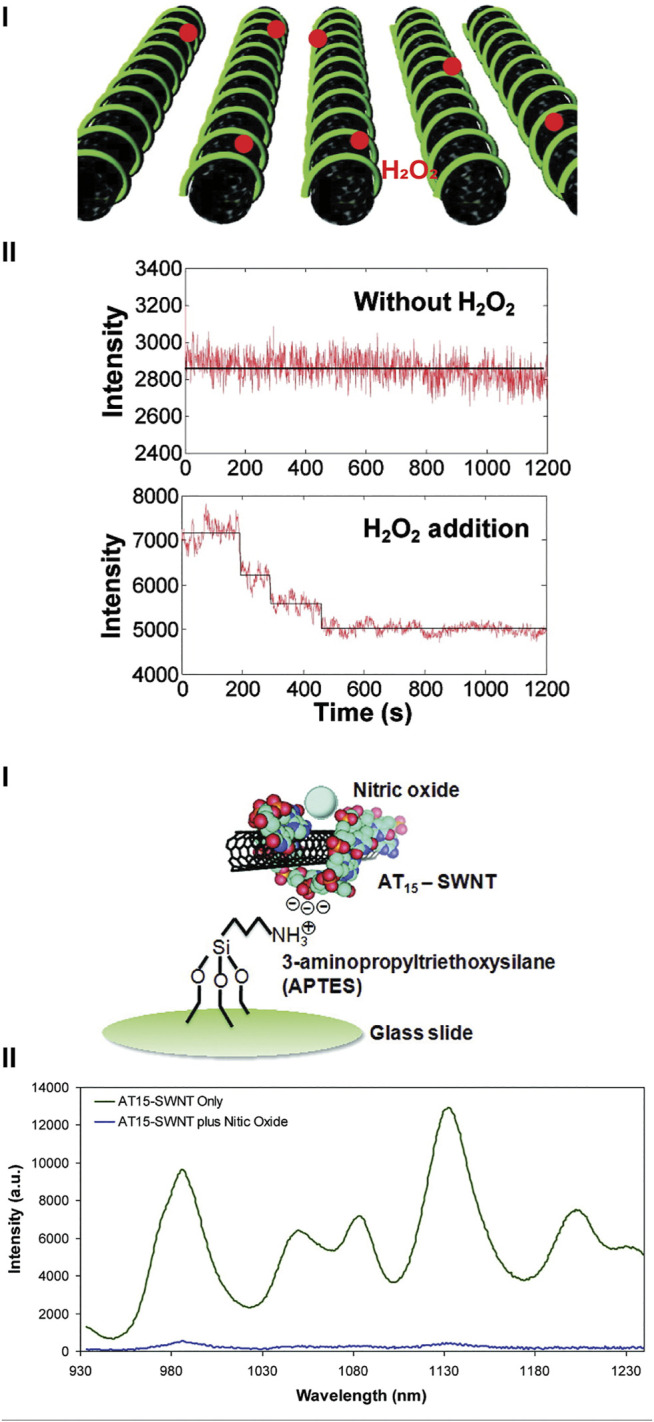
Single walled carbon nanotubes (SWCNTs) for detecting ROS and RNS. **[(A), i]**. SWCNTs coated with collagen for the detection of H_2_O_2_, where the presence of H_2_O_2_ changes the fluorescence emission. Reprinted (adapted) with permission from Kim et al. (2011). ACS Nano 2011, 5, 10, 7,848–7,857. Copyright 2011 American Chemical Society. **(B)** SWCNTs used for the detection of NO^•^. **[(B), i]**. DNA strand d(AT)_15_ was used to coat SWCNTs and immobilized onto an amine-functionalized glass slide. **[(B), ii]**. Fluorescence spectrum of d(AT)_15_-SWCNTs in the absence and presence of NO^•^. Reprinted (adapted) with permission from Zhang et al. (2011). J. Am. Chem. Soc. 2011, 133, 3, 567–581. Copyright 2010 American Chemical Society.

The Strano group has designed a wide range of ROS sensors using SWNCTs. Heller et al. (2009) developed a multiplex optical sensor for the detection of ROS *in vitro*. Here, SWCNTs were coated with a d(GT)_15_ oligonucleotide, in which depending on the type of ROS present, there was an apparent change in the fluorescence intensity and/or spectrum. Adsorption of H_2_O_2_ onto the surface decreased in the fluorescence intensity at both emission peaks (∼990 and ∼1040 nm), whilst in the presence of ^1^O_2_ there was a red-shift at the ∼990 nm peak emission spectrum, and if the nanosensor was in the presence of ^•^OH, there was a significant decrease of the peak emission at ∼1040 nm. From these three unique fluorescence changes, it was possible to interrogate real-time changes of H_2_O_2_, ^1^O_2_, and ^•^OH in 3T3 cells simultaneously following perfusion—demonstrating possible capacity for multiplex monitoring. Other examples from Strano group are shown in Figure 4, where they developed nanosensors for H_2_O_2_ (Kim et al., 2011) and NO^•^ (Zhang et al., 2011).

Recently, Safaee and co-workers (Safaee et al., 2021) developed a wearable optical nanosensor to monitor hydrogen peroxide levels as an inflammation biomarker. It has been reported that hydrogen peroxide acts as a signaling molecule during an inflammatory response to recruit cells for wound healing (Roy et al., 2006; Loo et al., 2012). In this work, SWCNTs were wrapped with (GT)_15_ and suspended within microfibres through coaxial electrospinning. This produced a wearable optical sensor for real-time monitoring of inflammation and wound healing. The SWCNTs were retained within the microfibrous network for up to 21 days, with no evidence of the nanosensors diffusing out of the 3D-scaffold. Furthermore, the scaffold was able to display spatial detection of hydrogen peroxide within a wound surface. The group was able to integrate this fabricated optical microfibrous nanosensor into existing wound bandages whilst maintaining the optical signal output.

#### Innate Fluorescent Carbon Dots for ROS Detection and Monitoring

Following their discovery in 2004 (Xu et al., 2004), carbon dots have been widely explored as fluorescent probes for the detection of metal ions and small molecules, including ROS (Sun and Lei, 2017). As a platform for optical biosensing, carbon dots possess tunable fluorescence properties through elemental doping (Feng and Qian, 2018), and tunable surface functional groups, depending on the carbon source and synthesis method. When compared to other well-established fluorescent nanoparticles, such as dye-functionalized particles or quantum dots, the use of carbon dots as biosensors appear to be biocompatible and do not contain heavy metals or other known toxins (Song et al., 2014b).

**FIGURE 5:**
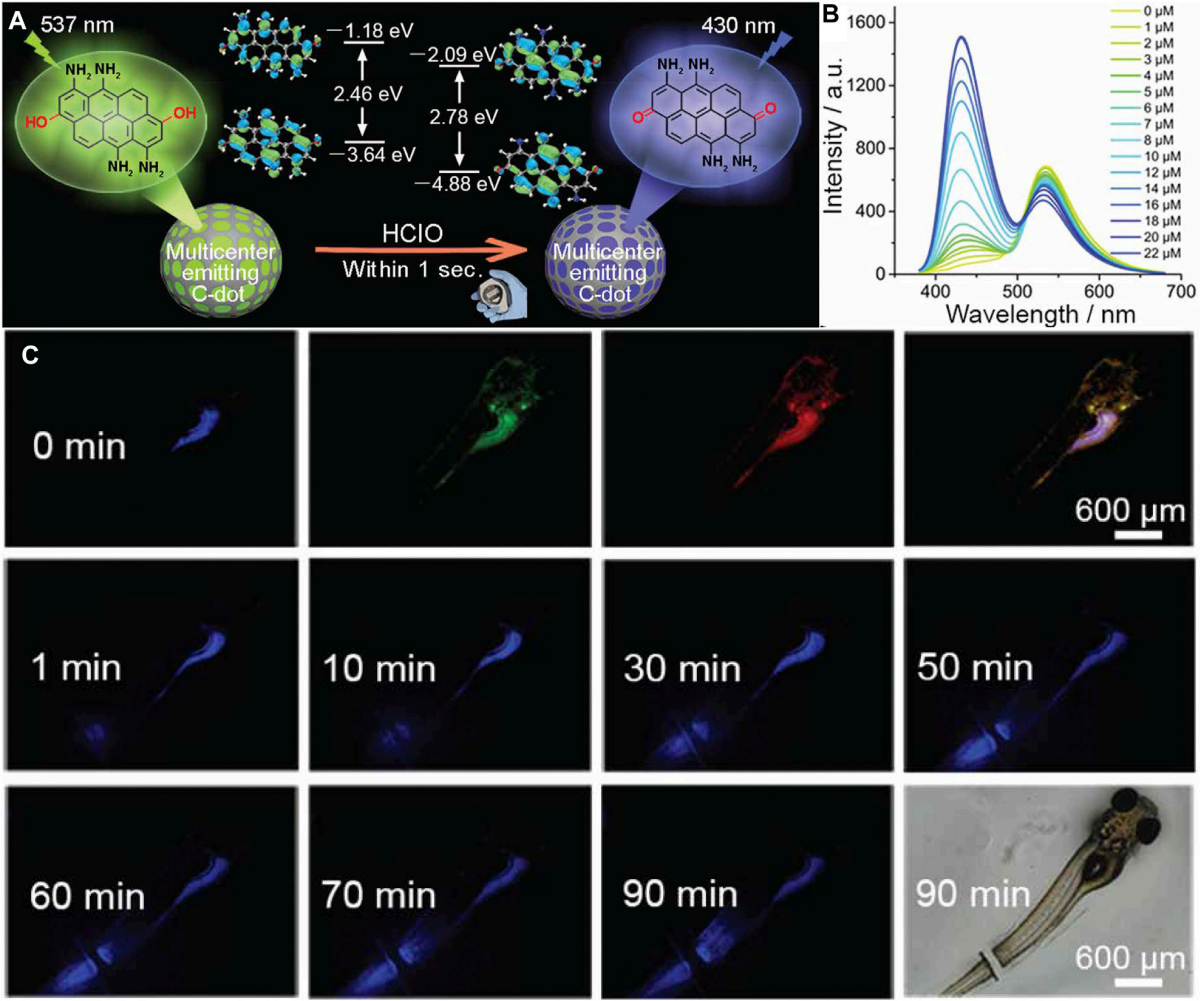
**(A)** Schematic of the “multicenter-emitting” carbon dot for the detection of ClO^−^. Here, due to the differences in energy state presence within the carbon dot, a ratiometic sensor was designed. **(B)** Fluorescence spectrum of the carbon dot at difference concentrations of ClO^−^, highlighting the change of peak emission from 537 to 430 nm. **(C)** Real time monitoring of *in vivo* wound healing on a zebrafish model using carbon dots. At 0 min, a representative fluorescence images of the zebrafish under blue, green, and red excitation were recorded pre-wound. Following amputation, fluorescence within the blue channel increased over a 90-min period, signifying increased levels of ClO^−^. Reprinted (adapted) with permission from Wang et al. (2020b). Chem Mater. 2020, 32, 19, 8,146–8,157. Copyright 2020 American Chemical Society.

Work by Wu et al. (2017) highlighted the feasibility of using carbon dots to detect ONOO^−^ within the mitochondria of living cells. By using phenylenediamine as their carbon source, the authors were able to synthesize 7 nm carbon dots with amine groups. The presence of amine groups served three purposes: 1) allowed for their sensors to be easily internalized; 2) the surface could be further modified with a mitochondria-targeting moiety, and 3) the oxidation of the amine groups by ONOO^−^ changes the fluorescence properties of the carbon dot. When uptaken by MCF-7 cells, the authors were able to confirm that their nanoprobes were internalized, while showing dynamic changes in ROS levels once an external stimulus was applied. In another study, Wang et al., 2020b demonstrated the use of carbon dots as *in vivo* fluorescent biosensors for ClO^−^ within zebrafish (Figure 5A). Here, a ratiometric “multicenter-emitting” nanosensor was developed by using m-aminophenol as their base material, where the selective presence of ClO^−^ would create a blue shift in the fluorescence spectrum, from 537 to 430 nm (Figure 5B). To demonstrate the functionality of their nanosensor *in vivo* to detect the presence of ClO^−^ in both digestive and metabolic systems, and during a wound healing response. It has been widely reported that an increased production of ClO^−^ (Wang et al., 2020a) and other ROS molecules occur during wound healing (Niethammer et al., 2009; Love et al., 2013). Through facile feeding, the authors were able to track the production of ClO^−^ within the intestines, where there was a sharp increase in fluorescence signal after 10 min. When used as a sensor for wound healing, the authors were able to visualize increased ClO^−^ associated with healing, due to the increased fluorescence signal near the wound site (Figure 5C), highlighting future applications as diagnosis tools.

#### Surface-Enhanced Raman Scattering Nanoparticles for ROS Monitoring

Raman scattering is a photophysical phenomenon where a small fraction of light is inelastically scattered from a surface. The amount of inelastic scattering can be used to quantify the analyte concentration on the surface. However, the low efficiency of light scattering means that Raman scattering has been limited to samples in high concentration (Sackmann and Materny, 2006). The use of nanoparticles has been found to significantly enhance the signal whereby molecules on the magnitude of parts per billion (ppm) can be detected (Kneipp et al., 1999). This phenomenon is referred to as surface-enhanced Raman scattering (SERS). Commonly, metallic nanomaterials, such as gold (Peng et al., 2016; Kumar et al., 2017) and silver (Shen et al., 2019) nanoparticles, have been used to detect reactive oxygen species, where changes within the intensity of the Raman spectrum can be used to correlate with analyte concentration.

Peng et al. (2016) developed a ratiometric SERS nanosensor to detect H_2_O_2_ within living cells and cancerous tissue. A gold nanorod was coated with thiol-functionalized phenylboronic ester which reacted with H_2_O_2_, causing a decrease in the signal at 993 cm^−1^ while leaving the Raman band intensity at 1,071 cm^−1^ remain unchanged. When incubated with either HeLa cells or within *ex vivo* cervical tumor models, cells and tissue treated with H_2_O_2_ could be clearly identified via a reduction in the Raman signal intensity at 993 cm^−1^. Further treatment with a ROS inhibitor, N-acetylcysteine, reduced the abundance of H_2_O_2_, and there was a subsequent increase signal intensity at 993 cm^−1^. Similarly, Chen et al. (2018) used a gold nanoparticle coated with thiol-functionalized phenylboronic ester to detect ONOO^−^ within macrophages. In this instance, the characteristic Raman shift at 882 cm^−1^ for ONOO^−^, compared to a constant signal at 993 cm^−1^ to develop a ratiometric sensor. Incubating probes with macrophages and concurrently simulating an immune response, the authors were able to track endogenous ONOO^−^ production.

## Emerging Technologies for ROS and Oxygen Monitoring *in vitro*

### Dynamic and Reversible Fluorophores and Nanosensors for Continuous ROS Monitoring

One of the main drawbacks of ROS-detecting nanosensors is that their application for continuous and dynamic monitoring is hampered by the fact that the commonly available ROS-sensitive dyes use irreversible chemical and/or structural changes for sensing. Identifying suitable reversible dyes will be a critical step for clinical utility of such sensors, which will require continuous and dynamic monitoring of ROS levels to effectively detect and monitor disease progression or treatment effects. Recently, selenium-doped fluorophores have been shown to reversibly monitor ROS due to the redox properties of the metals. This is based on the structure of glutathione peroxidase (GPx), where the selenol group on selenocysteine can undergo a reversible reaction with hydrogen peroxide (Rotruck et al., 1973). Since first being reported by Miller et al. (2007) for diagnostic applications, many groups have worked to improve selenium and tellurium-doped fluorophores for similar applications.

**FIGURE 6:**
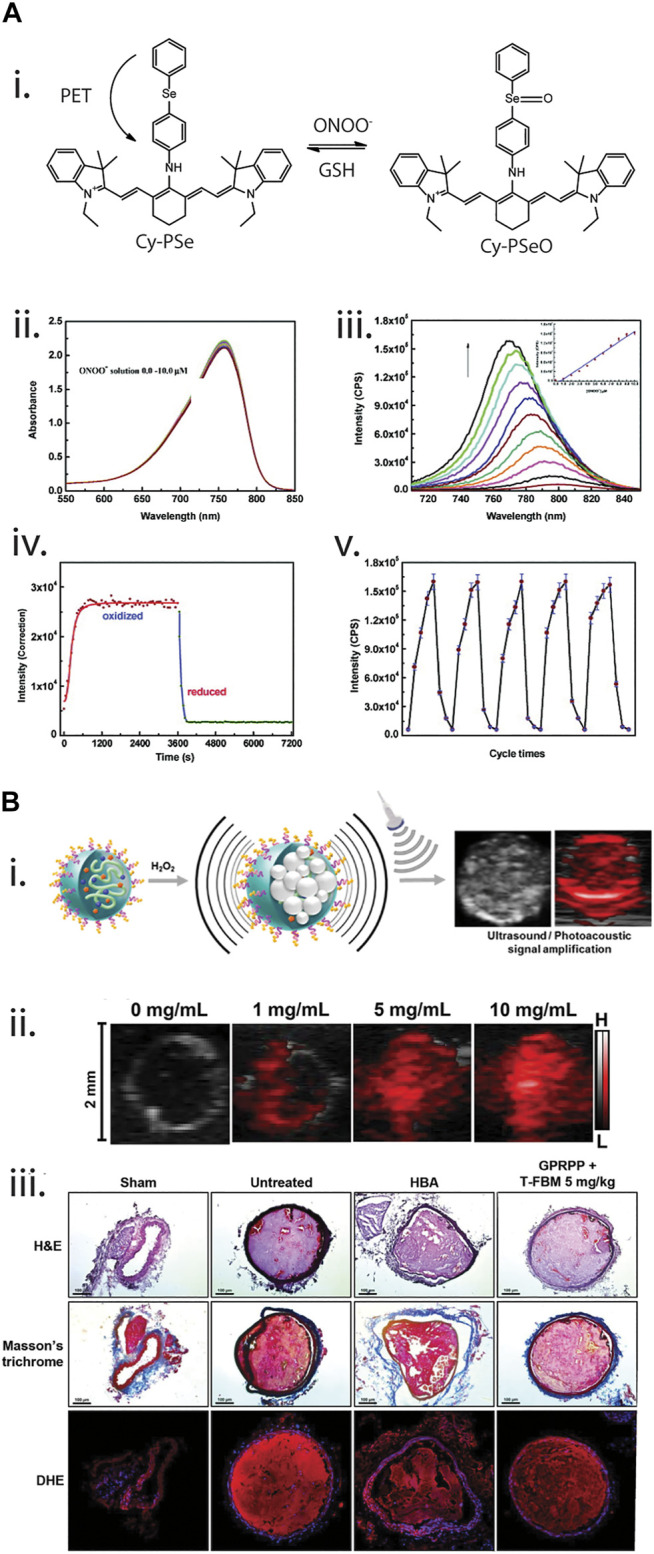
**(A)** Near-infrared cyanine-derived fluorophore for dynamic monitoring of peroxynitrite (ONOO^−^). **[(A), i]**. Reaction pathway of the fluorophore, where initially it is non-fluorescent due to a photoinduced electron transfer (PET) between the cyanine structure and the phenylselenyl group. However, once oxidized by ONOO^−^, the structure can fluoresce. In the presence of glutathione (GSH), the oxidized phenylselenyl group could be reduced and quenched the fluorophore. **[(A), ii,iii]**. Absorbance and fluorescence spectrum of the fluorophore at different concentration of ONOO^−^. **[(A), iv,v]**. Reversibility studies of the fluorophore under one and multiple cycles. Reprinted (adapted) with permission from Yu et al. (2011). J. Am. Chem. Soc. 2011, 133, 29, 11030–11033. Copyright 2011 American Chemical Society. **(B)** Theranostic photoacoustic nanosensors (T-FBM) for detection and treatment of thrombosis. **[(B), i]**. Detection of H_2_O_2_ using photoacoustic-responsive nanosensors, where the photoacoustic signal changes in the presence of H_2_O_2_. **[(B), ii]**. Photoacoustic signal of the nanosensors under different concentrations of H_2_O_2_. **[(B), iii]**. Therapeutic effects of T-FBM nanosensors, where thrombosis was minimised with the nanosensors when coated with thrombi-targeting lipopeptide (GPRPPC). Reprinted (adapted) with permission from Jung et al. (2018). ACS Nano 2018, 12, 1, 392–401. Copyright 2018 American Chemical Society.

The Han group has developed a wide range of probes to dynamically monitor ROS *in vitro*. In their initial work, Yu et al. (2011) developed a near-infrared fluorescence probe to detect peroxynitrite (Figure 6A). The use of a modified near-infrared cyanine dye with a phenylselenyl group (Cy-PSe), allowed the design of a reversible dye where the phenylselenyl group quenched the fluorescence of the cyanine dye. When the selenium group was in its oxidized state, however, a fluorescence signal was emitted. This reversible probe was used to monitor changes in ONOO^−^ levels in macrophages via imaging fluctuations of ONOO^−^ with cyclic loading of 3-morpholinosydnonimine (SIN-1) and glutathione S-transferase. Additionally, Lou et al. (2013) synthesized a diselenide-doped fluorescein dye (FSeSeF) for the visualization of intracellular glutathione. Glutathione has been widely reported as an antioxidant agent that maintains ROS levels *in situ*. When FSeSeF was in the presence of glutathione, the diselenide bond was cleaved, producing a strong fluorescence signal. This deselenium bond could be reform to allow the monitoring of dynamic changes in glutathione and H_2_O_2_ concentration. By staining HeLa cells and treating the cells with H_2_O_2_ and α-lipoic acid, a promoter for glutathione activity, it was demonstrated that this produced detectable and reversible changes in the fluorophore signal. Recently, Li et al., 2020b demonstrated that selenium-modified dyes can be used to dynamically monitor ClO^−^. Here, fluorescein was used as the base dye, where the specific attachment of the selenide group allowed the dye to selectively respond to ClO^−^. In order to demonstrate the functionality of the dye *in vitro*, Li and others exposed HL-60 cells to H_2_O_2_, stimulating induction of apoptosis. Incubation of the dye with cells, allowed demonstration that show ClO^−^ production was linked to the loss of mitochondrial membrane potential and apoptosis.

More recently, tellurium-doped fluorophores have also been explored as an alternative metal in the design of reversible ROS dyes. Compared to selenium dyes, the presence of tellurium can increase the sensitivity of reversible ROS dyes, due to lower electronegativity of the metal (Fang et al., 2015). Moreover, tellurium has been reported to be less cytotoxic than selenium (Engman, 1985), making it highly attractive for further development. Unfortunately, the use of tellurium-based ROS dyes has not been explored extensively, possibly due to the poor chemical stability of organotellurium (Engman, 1985). Work by Manjare et al. (2014) exploited the similarities and differences between selenium and tellurium-based BODIPY fluorophores for ROS sensing, where the tellurium-based dye showed a faster response and sensitivity compared to selenium counterparts.

Koide et al. (2012) developed a near-infrared fluorescence stain for monitoring ROS by synthesizing 2-Me TeR, a rhodamine-based dye modified with a tellurium group, which showed reversible fluorescence in the presence of ^•^OH, ONOO^−^, and OCl^−^. Initially, the dye is non-fluorescent, however, when in the presence of these three species, it forms the fluorescent compound 2-Me TeOR. This sensor showed dynamic capabilities *in vitro* by incubating with HL-60 cells. HL-60 cells express high levels of OCl^−^ when exposed to H_2_O_2._ These sensors were able to monitor the production and subsequent reduction of OCl^−^ as cells treated with H_2_O_2_ responded and subsequently returned back to homeostasis. Further demonstration of the dynamic nature of the sensor involved dosing of the cells with additional H_2_O_2_, which gave an increase and subsequent decrease in the fluorescence signal of the probe, demonstrating the reversible nature of the dye.

While the above section discussed the development of reversible fluorophores for ROS monitoring, there have been few reported cases of incorporation of these fluorophores into nanomaterials. The Tang group investigated the possibility for the development of nanosensors using selenium-based fluorescence probes for peroxynitrite in their initial work by Xu et al. (2011), which developed a reversible near-infrared fluorescence dye: benzylselenide-tricarbocyanine (BzSe-Cy). The fluorescence properties of BzSe-Cy are quenched due to the oxidation of the selenium. This reaction could be reversed in the presence of the reducing agent, ascorbate, resulting in reduction of the oxidized selenium. Following on from this, Tian et al. (2011) *.* incorporated BzSe-Cy into a ratiometric polymeric nanosensor for the detection peroxynitrite. By using isopropyl rhodamine B as a reference dye and using an amphiphilic block copolymer with cell penetrating peptides moieties, the authors were able to create micelle nanosensors for peroxynitrite monitoring. They also demonstrated the practical application of their probe *in vitro* by encapsulating the sensors within macrophages for intracellular imaging. Once the cells were exposed to SIN-1, a peroxynitrite donor, the authors observed quenching of the fluorescence signal. They also demonstrated the specificity of their sensor by exposing cells to other reactive nitrogen/oxygen species. Extension of this work could provide future opportunities for the development of reversible ROS-based nanosensors.

#### Photoacoustic Imaging for Non-Invasive, Deep Tissue Detection and Monitoring for ROS

A key limitation of optical nanosensors is their poor tissue penetration within the visible light spectrum, mainly associated with tissue autofluorescence and light scattering. While near-infrared fluorescence probes and sensors are being developed to specifically address this issue, the maximum depth penetration is still only on the length scale of millimeters, severely limiting their use in applications for which deeper imaging into the tissue is required. One alternative is photoacoustic (PA) imaging (Lee et al., 2019), which has a tissue penetration depth in the magnitude order of centimeters (Kim et al., 2010a). Imaging by photoacoustics relies on the interaction of near-infrared light with a contrast agent. Here, the incoming energy from the laser is absorbed by the contrast agent and acoustic waves are generated via thermal expansion of the contrast agent which can be detected through a sonograph. Common nanomaterials used in developing photoacoustic probes are single-walled carbon nanotubes, semiconductive polymers, and gold nanoparticles (Upputuri and Pramanik, 2020).

One prominent group that has developed photoacoustic probes and sensors is the Pu group. In their initial work, Pu et al. (2014) reported an *in vivo* photoacoustic nanosensor to detect ROS in mice. Here, the authors developed a ratiometric photoacoustic nanosensor that used a semiconducting polymer as a photoacoustic contrast agent/scaffold and attached a ROS-reactive dye (IR775S) for ROS detection. As a result of nanoencapsulation of the dye in their device, there was a higher selectivity towards ONOO- and OCl-when compared to the free dye, suggesting that nanostructure helped discriminate ROS molecules with shorter lifetime. To demonstrate the advantages of photoacoustics for deep tissue imaging, the sensors were injected intramuscularly into the thigh for acute oedema. By simulating the production of ROS *in vivo*, it was possible to visualize inflammatory ROS generation confirming these sensors a useful tool for deep tissue ROS imaging.

Similarly, Zhang et al. (2017a) developed a ratiometric photoacoustic sensor for the detection of ONOO^−^ *in vivo.* This used a boronated-caged boron-dipyrromethene dye as the ROS-reactive dye which was encapsulated within a semiconductive polymeric scaffold with triphenylborane to improve ONOO^−^ selectivity. The developed sensor used peak wavelength shifts to quantify ROS levels, where the dye had an absorbance peak at 675 nm. In the presence of ONOO^−^, a shift in the peak wavelength to 745 nm was observed and this was attributed to the rapid oxidative cleavage of the boron-dipyrromethene. Therefore, the ratio between the intensity at 745 and 675 nm permitted quantification of the relative amount of ROS present. To demonstrate the functionality of the photoacoustic sensors *in vivo*, the sensors were subcutaneously injected into mice tumor models and the PA signal at 750 and 680 nm were measured. Due to the presence of ONOO^−^ present within the tumor, the authors were able to visualize the dynamic changes in ROS levels over a 24-h period.

Photoacoustic sensors have also found utility in monitoring of cardiovascular conditions such as thrombosis. Jung and co-workers (Jung et al., 2018) designed a theranostic nanomedicine to detect thrombosis by conjugating borylbenzyl carbonate and the near infrared dye IR780 to maltodextrin (termed as FBM nanoparticles) (Figure 6B). The diagnostic/imaging capability stemmed from the ability of T-FBM nanoparticles to target the thrombus, a complex network of platelets and water-insoluble fibrin, which is accompanied by H_2_O_2_ generation during platelet activation, *via* functionalization of FBM nanoparticles with a thrombus-targeting lipopeptide known as GPRPPC. In the presence of H_2_O_2_, oxidation of aryl boronate occurs, leading to a chain of reactions and ultimately generating CO_2_ bubbles that significantly amplified photoacoustic signals in a mouse model of FeCl_3_-induced arterial thrombosis. The technique of enhancing photoacoustic signals hold significant advantages over conventional photoacoustic vaporization-based photoacoustic imaging with photoabsorber-containing nanodroplets as it does not rely on gas precursors (such as perfluorocarbon) and an external pulsed laser. The therapeutic functionality of these nanoparticles stems from the production of antioxidants and anti-inflammatory hydroxybenzyl alcohol (HBA) *via* quinone methide that is also a product of the oxidation reaction of aryl boronate in the presence of H_2_O_2_. While the study highlights the potential of this nanomedicine to serve as a theranostic agent for thrombosis, the limited penetration depth of IR780 could serve as an obstacle for its translation towards testing in clinical trials.

Yang et al. (2018) demonstrated the potential of combining photoacoustic imaging with active drug treatment by encapsulating cisplatin, a well-known platinum-based cancer drug, the ROS-sensitive IR790s and chelated ferric ions within a self-assembled polymeric scaffold. When exposed to the tumor microenvironment, cisplatin dissociates from the nanosensor and generates H_2_O_2_ and superoxide from O_2,_ where the former further reacts with ferric ions to form ^•^OH. The presence of ^•^OH was detected by IR790 where a photoacoustic ratio measurement could be obtained by the signal from 790 to 680 nm. These nanoparticles were shown to successfully target xenografted U87MG tumours in mice with, yielding a distinct photoacoustic signal only in the presence of nanoparticles supplemented with chelated ferric ions. This demonstrates the importance of Fe^3+^ in generating ROS signals for photoacoustic imaging.

#### Moving Away From the Visible Light Region: Emerging NIR-II Fluorescent Contrast Agents

Near infrared-II (NIR-II) contrast agents have been explored as fluorescence contrast agents for oxygen and reactive oxygen species. As highlighted previously, the main limitation of conventional fluorescence probes for oxygen and ROS is that they are confirmed to the visible spectrum (380–750 nm) (Cao et al., 2020), thus limiting imaging due to tissue scattering, poor depth penetration, and tissue autofluorescence. Imaging within the near infrared region (700 + nm) has mitigated these shortcomings due to the effective attenuation coefficient of tissue components (lipids, skin, and blood), which are relatively low (Smith et al., 2009). While NIR-I (700–950 nm) is adequate for deep tissue imaging, the light penetration depth is limited to 1–2 cm (Bashkatov et al., 2005), whereas NIR-II (1,000–1,300 nm) is more promising for non-invasive sensing due to a maximum of 4 cm tissue light penetration (Bashkatov et al., 2005).

Recently Zhao et al. (2020) developed a ROS-responsive FRET nanosensor to ONOO^−^ within a carcinoma tumor model. By pairing a NIR-II cyanine dye, MY-1057 with a Nd+3 doped/lanthanide nanoparticle, acting as a FRET pair, it was possible to differentiate between tumors and healthy tissue through fluorescence lifetime analysis. Lifetime measurements within the NIR-II region provide better resolution for deep-tissue imaging, as fluorescence imaging is limited due to high signal attenuation and tissue light scattering effects. (Hong et al., 2017; Fan et al., 2018). This advantage of NIR-II and lifetime measurements mean that it was possible to conduct deep-tissue imaging *in situ* in tumor bearing mice. Lifetime imaging enabled quantification of ONOO^−^ levels up to a 5 cm tissue penetration depth, highlighting the possibilities of non-invasive imaging with minimal signal attenuation.

## Outlooks and Conclusion

Oxygen and ROS are key candidates for cellular monitoring for research and clinical applications due to their links to biological reactions and disease progression. While hypoxia and subsequent ROS generation have been linked to countless diseases including cancers, neurological disorders, and cardiovascular disease, technologies to support spatially resolved, non-invasive and real-time imaging of oxygen and ROS has been limited. Although fluorescence-based techniques exist, such as commercially viable stains and fiber-optic probes, these techniques are unable to accomplish these criteria—stemming from their innate chemical structure, limited detection zone, or “end-point” quantification. Optical nanosensors have emerged as an opportune technology to meet the challenges of monitoring ROS and oxygen levels *in situ*. Many studies report using existing and commercially available dyes and encapsulating them within scaffolds to improve their optical properties or enhance their cellular uptake. This approach has yielded both FRET-based nanosensors and dye-labelled sensors that can monitor ROS and oxygen levels non-invasively, and in some cases have even been used to track disease progression. However, these sensor designs are still hindered by the chemical limitations of commercially available optical stains for oxygen and ROS, specifically the lack of dynamic and reversible monitoring, making them non-ideal nanosensors—as per Clark and Ruckh classification.

To better understand oxygen and ROS, and their connection with disease progression, emerging optical technologies based on reversible selenium and tellurium fluorophores have shown promise as a means to visualize dynamic changes in ROS levels *in situ*. While this field is still in its infancy, the studies in this area suggest great promise for these to generate improved understanding of the dynamic changes of ROS in biological systems both *in vitro* and *in vivo*. Moreover, work to improve the spatial resolution of ROS and oxygen localization *via* photoacoustic and NIR-II dyes has shown great promise in better depth profiling, where greater understanding of disease development can aid in potential and future therapy.

Overall, the field of fluorescence nanosensors show great potential to revolutionize the spatio-temporal monitoring of both oxygen and ROS. Building upon recent key advances in reversible dyes and methods for improved imaging within complex 2D/3D cell cultures and biological tissue, it is likely that these will make significant impact to our understand of the role of oxygen, ROS in biological processes and our ability to monitor these for clinical application, or improved development of therapeutic agents.
